# How do general practitioners and specialists value their mutual communication? A survey

**DOI:** 10.1186/1472-6963-9-143

**Published:** 2009-08-08

**Authors:** Annette J Berendsen, Annegriet Kuiken, Wim HGM Benneker, Betty Meyboom-de Jong, Theo B Voorn, Jan Schuling

**Affiliations:** 1 Department of General Practice, University Medical Centre Groningen, University of Groningen, Ant. Deusinglaan 1, 9713 AV Groningen, The Netherlands; 2 Department of Primary Care, University Medical Centre Saint Radboud, University of Nijmegen, The Netherlands

## Abstract

**Background:**

Communication between general practitioners (GPs) and specialists is important, if we want patients to receive the right type of care at the right moment. Most communication takes place through telephone contact, letters concerning information on patients more recently also by email, and joint postgraduate training. As much research has been aimed at the content of communication between GPs and specialists, we wished to address the procedural aspects of this communication.

We addressed the following research question.

How do GPs and specialists assess their mutual communication through telephone, letters and postgraduate courses?

**Methods:**

A cross-sectional study was conducted among a random sample of 550 GPs and 533 specialists selected from the Netherlands Medical Address Book. The response rate was 47% GPs (n = 259) and 44% specialists (n = 232).

**Results:**

Specialists qualify the GPs' telephone accessibility as poor; while GPs themselves do not. Specialists think poorly of the GPs' referral letter. Merely half of GPs feels their questions are addressed appropriately by the specialist, whereas specialists think this number is considerably higher. According to specialists, GPs often do not follow the advice given by them. GPs rate their compliance much higher. Less than a quarter of GPs feel the specialist's letter arrives on time. Specialists have a different perception of this.

Both parties wish to receive feedback from one and other, while in practice they do so very little.

**Conclusion:**

GPs and specialists disagree on several aspects of their communication. This impedes improvements. Both GP's accessibility by phone and time span to the specialist's report could be earmarked as performance indicators. GPs and specialists should discuss amongst themselves how best to compose a format for the referral letter and the specialist's report and how to go about exchanging mutual feedback.

## Background

Communication between general practitioners (GPs) and specialists is important, if we want patients to receive the right type of care at the right moment [1]. Most communication takes place through telephone contact, letters concerning information on patients more recently by email, and joint postgraduate courses.

The GP uses the telephone to different ends: to consult a specialist, to arrange an emergency referral for the patient or to transfer specific information regarding the patient. The specialist usually uses the telephone for this last purpose only.

Through the most typical means of communication, i.e. correspondence consisting of referrals to and fro, the GP and the specialist aim to transfer relevant information about a patient and thereby give the other the necessary information to provide the needed care [2].

Historically, specialists educated GPs in postgraduate courses, whereas these days they are more consultants than teachers. During these courses, the specialist informs the GP on the latest relevant developments in his professional area of expertise [3,4]. Also meetings are organized during which GPs and specialists talk about overlapping areas of work and collaboration. Besides knowledge transfer, getting to know each other is also important at these meetings.

Few studies have been conducted on the effectiveness of telephone consults [5,6]. Knowing each other seems important: it results in greater satisfaction about the telephone communication [5,7,8]. One of the most important communication errors is that GPs provide incomplete information [9].

Concerning correspondences specialists feel that GPs' referral letter often contains inadequate or incomplete information, and does not always include a specific question on the GP's part [10,11]. The specialist's report sometimes does not hold enough information [12,13] and sometimes is too detailed in content [14,15]. Different studies show that the specialist's report could be improved by structuring it [14-16]. Delayed arrival of this letter is the major difficulty of the specialist's report [6,12].

Giving each other feedback is an important tool to improve the communication between specialists and GPs. It might lead to improvements in the referral letters or makes referrals more focused [17]. Joint postgraduate courses offer GPs and specialists the opportunity to give feedback informally, e.g. by using case reports.

As much research has been aimed at the content of communication between GPs and specialists, we wished to address the procedural aspects of this communication.

We addressed the following research question.

How do GPs and specialists value their communication through telephone, letters and education?

The following tentative hypotheses from our qualitative research were tested [7,8]:

- both GPs and specialists are dissatisfied with mutual accessibility by telephone;

- specialists state that the quality of GPs' referral letters is often insufficient;

- many GPs state that specialists do not address the question(s) posed in their referral letter;

- many GPs state it takes too long for the specialist to send a letter in return;

- many specialists have the impression that GPs do not follow their advices;

- a majority of GPs and specialists is advocate for the development of a joint digital medical record;

- feedback is rarely given, though it is highly wanted;

- GPs wish to teach and learn from specialists;

- specialists wish to teach GPs.

## Methods

A cross-sectional study was conducted among Dutch GPs and specialists. The questions were formulated based on the data gathered in earlier qualitative, explorative research among GPs and specialists [7,8]. They could be answered on a five-point scale ranging from 'completely agree' to 'completely disagree' (five point scale). The questionnaire also comprised questions on respondent characteristics, such as age, gender, medical specialty, type of practice, length of practice experience, whether they were a trainer and type of employment. The questionnaire was presented to a number of key figures (GPs and specialists) in the Netherlands, and about a dozen test questionnaires were taken to assess the applicability of the questionnaire (comprehension, formulation, length of time).

In a pilot study the questionnaire was tested using a random sample of 148 GPs and specialists in the Netherlands. This led to some adaptations. The adapted version was presented to a random sample of 550 GPs and 553 specialists from the Dutch medical address book. Specialists rarely contacting a GP, such as nuclear physicians and anaesthesiologists, were not invited to participate. Before the questionnaire was posted each addressee received an announcement. Non-respondents were later reminded by letter. This whole procedure was repeated a month later for non-respondents.

Within the group of GPs and specialists respectively subgroup analysis was performed for age, gender, length of practice experience, office setting, medical specialty, and whether the respondent was a trainer or not. For ease of analysis, specialties were reduced to three broad groups: physicians, surgeons, and supporting specialists. Testing was done through non-parametric tests (Kruskall Wallis, Mann-Whitney test, Spearman's rho and Kendall's tau-b/c). A p-value < 0.05 was considered significant [18,19]. For ease of interpretation the frequencies in the tables concern the percentage of the combined answers 'completely agree' and 'agree'.

The ethics committee of the University Medical Centre Groningen studied our methods and declared legal assessment was not required.

## Results

### Respondent characteristics

The study was conducted in March through to September 2006. Of the included GPs, 47% (n = 259), and of the included specialists, 44% (n = 232) returned the questionnaire. The GPs' average age was 50 (sd 6.7) and the specialists' was 51 (sd 7.6). The male/female ratio, length of practice experience, type of practice, type of employment, and the distribution of specialties are listed in table 1 and 2[20,21]. Tables 3, 4 and 5 show the results of the questionnaire.

**Table 1:** Characteristics of respondents

	GP n = 264	National	Specialist n = 232	National
Mean age (sd)	50 (6.7)	47.4	51 (7.6)	41% > 50

Female (%)	33	34	21	26

Years of practice experience P50 (P25-P75)	20 (13–26)	*	16 (9–24)	*

Trainers (%)	38	*	22	*

**Employed in (%)**				
City area	46	43		
Semi-urban area	38	43		
Rural area	16	13		
				
University hospital			26	*
Leading general hospital			29	*
Peripheral hospital			45	*

**Type of practice (%)**				
Single handed	29	25		
Twin	30	30		
Health centre	41	45		
				
Outpatient department			20	*
Clinic			3.1	*
Both			77	*

**Type of employment (%)**				
Self-employed	85	90		
Paid employment	15	10		
				
Self-employed			47	50
Paid employment			53	50

* Data not available

**Table 2:** Distribution of medical specialties

	Respondents n = 232	Percentage	Registered Specialists percentage
Physicians	137	58.9	59.1

Surgeons	70	29.9	29.8

Supporting specialists	25	10.8	11.1

**Table 3:** frequency of telephone contact

**Question** On average, how often do you seek contact with a specialist/GP by telephone?	**GP** **%** **n = 264**	**Specialist** **%** **n = 232**
More often	36.9	21.5

Once a week	49.0	34.6

Once a month	12.9	29.8

Once in 3 months	0.8	12.7

Never	0.4	1.3

GP versus specialist p-value < 0.001

Surgeons less than physicians and supporting specialists (p < 0.001).

**Table 4:** questions and frequencies of answers

**Question**	**GP agree (%)** **n = 264**	**Specialist agree (%)** **n = 232**	**p-value**
**Telephone contact**			

Specialist/I can generally be easily reached for colleague consultation.	73.3	76.8	0.47

I/GP can generally be easily reached for colleague consultation.	85.3	32.8	<0.001

**Correspondence**			

Generally, the GP's referral letter is qualitatively good.		29.1	

The specialist/I answer(s) the question in the referral letter.	50	87.5	<0.001

The specialist's/my report back to the GP is generally sent back in a timely manner.	22.5	61.8	<0.001

Generally, the specialist's report is qualitatively good.	82.7		

Generally, I/the GP follow(s) the recommendations made by the specialist in his report.	92.2	49.5	<0.001

The information exchange between GP and specialist (for example on complex/chronic patients) could be improved by using a joint electronic medical file.	77.0	70.8	0.14

**Feedback**			

I appreciate feedback from the GP/specialist on my actions.	94.9	89.0	0.015

**Professional expertise**			

Specialists/I have to play an important part in increasing my/GPs' medical knowledge.	60.7	62.9	0.66

I/GPs have to educate specialists/me on the impact of epidemiological differences between primary and secondary care. Specialists: difference between trainers and non-trainers p = 0.046	45.1	36.2	0.056

Specialists/I have to educate me/GPs half yearly on their field of medical expertise to keep GPs' medical knowledge up to date.	44.4	61.6	<0.001

A certain specialist/I has(ve) to educate me/GPs regularly (special interest).	22.6	65.9	<0.001

Agree = completely agree + agree

**Table 5:** frequency of feedback

**Question** How often do you receive feedback on your actions from a specialist/GP on average?	**GP** **%** **n = 264**	**Specialist** **%** **n = 232**
Never	16.4	17

Once a year	29.3	28.6

Once every 6 months	27.3	21.5

Once every 3 months	20.7	20.5

More often	6.3	12.4

GP versus specialist p-value 0.22		

**Question** How often do you give feedback to specialists/GPs on their actions on average?	**GP** **%** **n = 264**	**Specialist** **%** **n = 232**

Never	15.6	9.5

Once a year	35.5	18.0

Once every 6 months	25.4	25.4

Once every 3 months	18.8	25.9

More often	4.7	21.2

GP versus specialist p-value < 0.001

### Contact by telephone

GPs more often sought contact with specialists than vice versa. This is a significant difference (p < 0.001). Surgeons sought contact by telephone less than physicians and supporting specialists (p < 0.001). About three quarters of GPs and specialists were satisfied by specialists' telephone accessibility. Telephone accessibility of the GP was considered fine by most of the GPs (85.3%). The specialists disagreed: a third (32.8%) of the specialists thought GPs can be well-reached by telephone (p < 0.001). The correlations between the frequencies of contact by telephone and the questions in table 4 vary for GPs from 0.000 to 0.125 and for specialists from 0.028 to 0.127.

### Correspondence

Less than a third of the specialists (29.1%) thought the GPs' referral letter was of good quality. Half of the GPs (50%) thought the specialist correctly addressed the question posed in the referral letter. More specialists (87.5%) thought they addressed this question correctly (p < 0.001). Less than a quarter of the GPs (22.5%) thought the specialist's report was sent back on time. Over half (61.8%) of the specialists thought this report was sent back within an appropriate time span. The difference is significant (p < 0.001). Most GPs (82.7%) thought the specialist's report was of good quality.

GPs and specialists disagreed on whether GPs adequately followed specialists' recommendations in the specialist's report. Almost all GPs stated they followed these correctly (92.2%), but half of the specialists agreed with this statement (49.5% – p < 0.001).

Both groups were advocates for introducing a joint digital medical record (77 and 70.8%), irrespective of their age.

### Feedback

GPs (94.9%) and specialists (89%) both appreciated getting feedback (p = 0.02). Nearly three quarters of both groups received feedback on their actions once in six months or less (GP 73%, specialist 67.1%). Little feedback was given: 76.5% of GPs gave feedback to the specialist once in six months or less. Half of the specialists gave feedback to GPs a similar number of times (52.9%). Specialists gave feedback to GPs significantly more often than GPs did to specialists (p < 0.001). The correlation between the frequencies of giving and receiving feedback was 0.475 for GPs and 0.466 for specialists.

### Professional expertise

Almost as many GPs (60.7%) as specialists (62.9%) would like specialists to take active part in increasing GPs' expertise. Less than half of GPs (45.1%) and over a third of specialists (36.2%) would like GPs to educate specialists on the meaning of epidemiological differences between primary and secondary care. In this, the difference between trainers and non-trainers is significant in specialists (p = 0.046): trainers found this type of education more appealing. For the GPs, no such difference between trainers and non-trainers was found (p= 0.478). Less than half of the GPs (44.4%) wished a certain specialist to educate them half yearly and keep their medical knowledge up to date. Specialists are more motivated in this (61.6% – p < 0.001). A fifth of the GPs (22.6%) would like to specialize in a certain medical field (special interest). Two thirds of the specialists (65.9%) were willing to collaborate in this (p < 0.001).

## Discussion

### Telephone contact

Specialists qualify the GPs' accessibility by telephone as poor, while GPs themselves disagree. GPs increasingly offer access to an extra phone line for consultation, but specialists may not be informed about this facility. Besides, most GPs probably do not realize that having such an extra line does not guarantee an immediate access to the doctor himself. GPs more frequently seek telephone contact than specialists, as they have more reasons to do so. Surgeons less frequently seek telephone contact with GPs than physicians; the reason for this may be that contextual factors are of less importance in the treatment of surgical patients. Whereas knowing each other results in greater satisfaction about the telephone communication in our study more contact by telephone did not lead to more positive answers regarding the accessibility by phone, correspondence, feedback or professional expertise [5,7,8].

### Correspondence

Specialists think poorly of the GPs' referral letter. Our qualitative research showed that GPs' opinions on this varied greatly [8]. Some GPs do not put a lot of energy into the referral letter, because they feel specialists do not address their question precisely. Other GPs consider the referral letter an important mode of communication, which therefore should be drafted with care. The current study shows that merely half of GPs feels their questions are addressed appropriately by the specialist whereas specialists think this number is considerably higher. This discrepancy has been found earlier as well [22].

Though this research shows that most GPs think the quality of the specialist's report is good, other research shows that the specialist's letter sometimes contains incorrect information or even lacks certain data [23]. According to specialists, the GP often does not follow the advice given by them. GPs rate their compliance much higher. Possibly, the advice is not given sufficient emphasis in the specialist's report, or it remains hidden to the GP in an extensive report. Other factors probably contribute to this effect, such as differences in task interpretation, views on the relevance of psycho-social contexts in patient treatment, as well as the varying functions of a specialist's report, which are hard to combine in a single letter. The specialist uses it for the archive, whereas for the GP it is the most important tool for information transfer on the treatment to be followed. Importantly, research has shown that adding an evidence-based summary of one sentence to the specialist's report increases GPs' follow-up of the advice [24].

GPs and specialists differ substantially in their opinion on how long it takes for the GP to receive the specialist's report. Less than a quarter of GPs feels it arrives on time. Patients are of the same opinion [25]. Specialists have a totally different perception. Keeping in mind the desired continuity of care, this is an excellent item for improving quality of care.

Currently, The Netherlands is in the process of developing a digital referring system (Care domain), initiated and financed by hospitals in general [26]. The focus lies on the format of the referral letter and on access to the appropriate specialist. Our earlier qualitative research showed that specialists mainly wish to focus on the quality of the referral, whereas GPs appreciate a qualitatively good referral back [8]. So far, not much attention has been given to the latter.

Co-ordinating organizations (Dutch College of General Practitioners and the College of Medical Specialists) should develop guidelines not merely for digital referrals, but for the referral letter and the specialist's report as well. In making regional agreements, the GP usually is not equipped to represent the profession as a whole [8], therefore national guidelines should be formulated which can be adapted to different regions.

A surprisingly large number of GPs and specialists advocate a joint digital medical record. In this research, no distinction was made between different purposes for such a joint medical file (access, options to add notes, joint journals). Further research should discriminate these options in closer detail.

### Feedback

Though both parties wish to receive feedback from one and other, practice shows they do so very seldom. In both groups there is a reasonably high correlation between the frequencies of giving and receiving feedback. Probably are for both activities similar skills required. Though we expected these skills might develop in the course of a professional career no differences were found between subgroups.

According to specialists, they give more feedback than GPs. This concurs with the findings in earlier research, which shows that specialists highly value giving feedback as a means to improve quality of care. GPs give preference to information from specialists above books or articles, and they appreciate education being directly linked to the clinic [23].

Our qualitative research shows that some specialists feel they (implicitly) give feedback in their letters [7]. As the need for feedback appears to be so great, possibilities for sharing it should be increased, for instance by reserving a few lines in correspondence specifically for feedback, and by offering courses on a regional level.

### Professional expertise

GPs and specialists agree that specialists should play an important part in postgraduate courses of GPs. Our qualitative research shows that GPs would like specialists to gain more insight into the working method and professional expertise of GPs. There is no great enthusiasm among GPs to educate specialists on the impact of epidemiological differences between primary and secondary care; most likely, the concept GPs would like to convey to specialists is not clear. GPs are probably merely interested in increasing specialist's comprehension of the GP's working method and circumstances. In turn, specialists show little interest in learning more about epidemiological differences between primary and secondary care. Though, specialist-trainers find education by GPs on these differences more appealing than specialist non-trainers.

GPs are less-inclined to follow a long term educational training of a certain specialist. This is understandable, as GPs have contact with many different specialists and such a time-investment would be disproportionate. Besides this, GPs are mainly interested in directly applicable knowledge [3].

Specialists are keen to educate GPs with a special interest. These GPs show enthusiasm for the concerned specialist's discipline for example ophthalmology, asthma, and COPD. Competition does not seem to be a factor in this. A quarter of GPs would like to get such training in order to register as GP with a special interest. Considering the number of practicing GPs in The Netherlands, this is quite a high number, which underlines the importance of this training. These courses should be made more accessible and their subject chosen on demand. Until now, most of them have been based on supply.

### Strength/weakness

Both parties were researched with the same questions, which makes comparison possible. It is important, after all, to assess if certain noted problems are experienced by the profession concerned as well. Of course, this study mentions reported behaviour and is not a registration or observation.

GPs and specialists' response rate (47% and 44% respectively) is somewhat low. On the other hand, the results on age, gender, length of practice experience, type of practice and employment are a correct representation of GPs and specialists in The Netherlands [20,21]. This is also true for the distribution of specialties. It also has to be kept in mind that this study was conducted in The Netherlands, where the GP functions as a gatekeeper between patient and specialist. Possibly our results cannot be applied in countries where patients have direct access to medical specialists.

## Conclusion

The differences in perception between GPs and specialists on the accessibility by phone of the GP, the content of the mutual correspondence and the time span of the specialist's report are new findings. Feedback is important but the extremely rare appliance is new.

GPs need to realize their accessibility for specialists by telephone should be improved. Regional meetings should have this topic on the agenda: the availability of information on special phone numbers for consultation between colleagues, including the time span the GP can actually be reached.

Specialists need to realize their letters often arrive too late. This should also be on the agenda of regional meetings.

Both GP's accessibility by phone and time till arrival of specialist's report could be earmarked as performance indicators.

For both referral letter and specialist's report, a digital format composed by both parties should be developed. Obviously within this format a clearly demarcated, prominent place should be reserved for the GP's question in the referral letter and for the specialist's answer and advice for treatment.

GPs and specialists should discuss how best to exchange feedback.

This study shows, that the difficulties in communication experienced by one party are not acknowledged by the other party involved. This is probably the reason why these problems, though well known for a long time, still are not solved.

## Competing interests

The authors declare that they have no competing interests.

## Authors' contributions

AJB, WHGMB, BMdJ and JS contributed to the design of this study. AJB was responsible for the day-to-day management. AK worked on the statistics and produced the first draft of the manuscript. All authors contributed to the write-up of this study. All authors read and approved the final manuscript.

## Pre-publication history

The pre-publication history for this paper can be accessed here:


